# Hormone replacement therapy does not increase thrombosis risk following THA: a national database study

**DOI:** 10.1186/s40634-023-00620-0

**Published:** 2023-06-01

**Authors:** Brian P. McCormick, Sean B. Sequeira, Mark D. Hasenauer, Henry R. Boucher

**Affiliations:** Department of Orthopaedic Surgery, MedStar Union Memorial Hospital, 3333 North Calvert Street, Suite 400, Baltimore, MD 21218 USA

**Keywords:** Total hip arthroplasty; Hormone replacement therapy, Thromboembolism, Postoperative complications

## Abstract

**Purpose:**

Hormone replacement therapy (HRT) causes a significant increase in the risk of venous thrombosis. The risk of medical and surgery-related complications among women taking HRT following total hip arthroplasty (THA) is poorly understood, and there are currently no guidelines in place regarding venous thromboembolism prophylaxis in this patient population. The purpose of this study was to evaluate the frequency of early medical and surgery-related complications following THA among women taking HRT.

**Methods:**

Women aged > 40 years of age who underwent primary THA were identified from a retrospective database review. A control group of non-HRT users was matched using propensity scoring to HRT users. Rates of 90-day medical complications and 1-year surgery-related complications were compared between cohorts using odds ratios. Postoperative anticoagulation regimens were also compared.

**Results:**

There were 3,936 patients in the HRT cohort who were matched to 39,360 patients not taking HRT. There were no significant differences in rates of DVT (OR 0.94, *p* = 0.6601) or PE (OR 0.80, *p* = 0.4102) between cohorts. Patients on HRT were more likely to sustain a dislocation (OR 1.35, *p* = 0.0269) or undergo revision surgery (OR 1.23, *p* = 0.0105). HRT patients were more likely to be prescribed warfarin (OR 1.21, *p* = 0.0001) or enoxaparin (OR 1.18, *p* = 0.0022) and less likely to be prescribed rivaroxaban (OR 0.62, *p* < 0.0001) compared to controls.

**Conclusions:**

HRT was not found to be an independent risk factor for thromboembolism following THA. Further research is warranted to better delineate the ideal perioperative medical management of HRT users undergoing THA.

## Introduction

Primary total hip arthroplasty (THA) is a commonly performed orthopaedic procedure with an expected volume of 635,000 cases per year by 2030 [23]. One of the most common medical complications following THA is the development of deep vein thrombosis (DVT), with reported incidence ranging from 0.2% to 0.59% [5, 14, 20]. Hormone replacement therapy (HRT) is widely used by women to treat symptoms related to menopause and has been shown to improve bone health through antiresorptive properties [3, 19]. HRT also exerts a thrombogenic effect by increasing levels of serum fibrinogen, factors II, VII, VIII, and X while decreasing levels of antithrombin and protein S [18, 22], and women taking HRT have been found to be at 2.7 to 3.6 times increased risk of idiopathic DVT formation [4–6, 8]. Younger patients are more frequently being considered appropriate candidates for THA [2, 9, 13], and patients taking HRT are typically treating symptoms related to menopause. Therefore, it should be expected that the prevalence of HRT users undergoing THA will increase. There is currently a paucity of literature regarding the risk of postoperative thromboembolism following THA among women taking HRT. The most recent clinical practice guidelines for patients undergoing elective THA from the American Society of Hematology do not include protocols specific to HRT patients [1].

HRT has a known effect on bone health through antiresorptive properties mediated through the nuclear factor kappa B ligand (RANKL) system [3, 19]. This may impact surgery-related complications and implant survivorship following arthroplasty procedures. It stands to reason that the antiresorptive properties of HRT may function similarly to bisphosphonates, which have been shown to reduce rates of periprosthetic bone loss, implant migration, aseptic loosening, and revision surgery [11, 16, 21]. A prior case–control study demonstrated a protective effect of HRT therapy against revision surgery following total joint arthroplasty [15], although further research investigating this relationship is lacking. Arthroplasty surgeons would benefit from a better understanding of surgery-related complications associated with HRT in order to accurately risk stratify patients prior to performing THA and to counsel patients appropriately.

Given the prevalence of HRT use among the arthroplasty population and the relative paucity of literature available, it is important to better understand how HRT alters the risk of medical and surgery-related complications following THA. This would allow proper counseling of patients in the preoperative period and may guide decisions regarding the discontinuation of HRT in the perioperative period or alter postoperative anticoagulation regimens. The purpose of this investigation was to evaluate the association between HRT use and postoperative complications among patients undergoing THA.

## Methods

This is a retrospective cohort study utilizing the commercially available Mariner database via PearlDiver (PearlDiver Inc., Colorado Springs, Colorado, USA). This database contains deidentified records for 151 million patients in the United States in accordance with the Health Insurance Portability and Accountability Act (HIPAA). Patient records were queried using International Classification of Diseases (ICD) and Current Procedural Terminology (CPT) codes. This study was deemed exempt from our institution’s review board process.

**Fig. 1:**
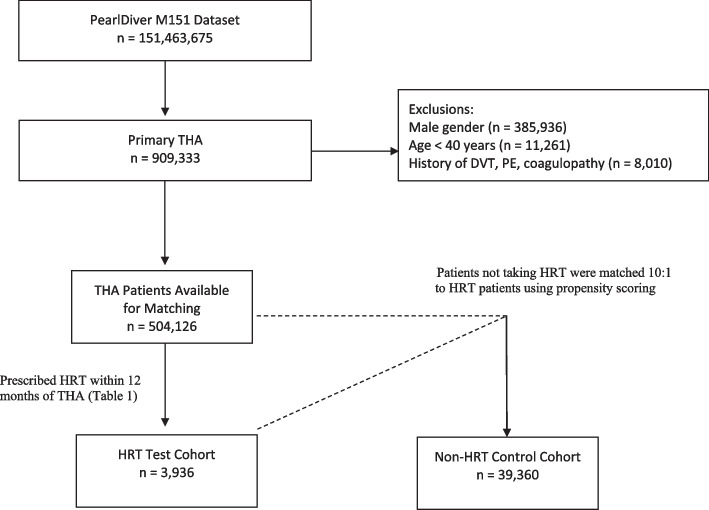
Flowchart of patient inclusion and exclusion criteria queried from the PearlDiver Mariner database

Female patients 40 years of age or older who underwent primary THA were identified using CPT and ICD codes. Patients with a history of DVT or pulmonary embolism (PE) prior to their index procedure and patients previously diagnosed with a hypercoagulable state (ICD-9-D-28981, ICD-9-D-28982) were excluded. Patients who were taking HRT at the time of surgery were identified using records of filled prescriptions within 12 months of surgery (Table 1). Propensity score matching was used to match patients taking HRT 1:10 to a control group of patients not taking HRT based on age, history of alcohol abuse, diabetes, obesity, hypertension, hyperlipidemia, renal disease, pulmonary disease, coronary artery disease, peripheral vascular disease, and tobacco use disorder (Fig. 1).

**Table 1:** Pharmacy insurance claims

Hormone Replacement Therapy	GENERIC_DRUG-DROSPIRENONE/ESTRADIOL, GENERIC_DRUG-ETHINYL_ESTRADIOL/DROSPIRENONE, GENERIC_DRUG-DESOGESTREL-ETHINYL_ESTRADIOL, GENERIC_DRUG-DROSPIR/ETH_ESTRA/LEVOMEFOL_CA, GENERIC_DRUG-ETHYNODIOL_D-ETHINYL_ESTRADIOL, GENERIC_DRUG-LEVONORGESTREL-ETHIN_ESTRADIOL, GENERIC_DRUG-LEVONORGESTREL/ETHIN.ESTRADIOL, GENERIC_DRUG-ESTRADIOL/LEVONORGESTREL, GENERIC_DRUG-NORGESTIMATE-ETHINYL_ESTRADIOL, GENERIC_DRUG-ESTRADIOL/NORGESTIMATE

Pharmacy claims data were queried to identify patients taking HRT prior to surgery. Patients were placed in the test cohort if they were prescribed any of these drugs within 12 months of undergoing primary THA

Ninety-day incidences of medical complications including DVT, PE, transfusion, urinary tract infection (UTI), pneumonia, and acute kidney injury (AKI) were evaluated and compared between cohorts. One-year surgery-related complications included dislocation, periprosthetic fracture, loosening, periprosthetic joint infection (PJI), all-cause revision surgery, and wound disruption. All-cause revision surgery was subclassified into femoral and/or acetabular component exchanging (ICD-9-P-0070, ICD-9-P-0072, CPT-27090, CPT-27091, ICD-9-P-8153, CPT-27134, CPT-27138, ICD-9-P-8005, CPT-27137) and component retaining (ICD-9-P-0073, CPT-26990, CPT-27030, CPT-27033, CPT-26991, CPT-27301) groups. Postoperative anticoagulation regimens were also evaluated and compared since more potent anticoagulation regimens would be a significant confounder while comparing DVT rates between cohorts. To maintain patient anonymity, the PearlDiver database does not report outcomes with fewer than 10 patients. Medical complications with low incidences such as myocardial infarction, sepsis, and cerebrovascular accident were therefore not able to be reported as outcome measures in the current study. Odds ratio (OR) and 95% confidence intervals (CI) were calculated for each variable independently using R (University of Auckland, New Zealand). A p-value less than 0.05 was considered statistically significant.

## Results

3,936 patients taking HRT prior to THA were matched 1:10 using propensity scoring to 39,360 patients not taking HRT with demographic and comorbidity data presented in Table 2. There were no significant differences in rates of 90-day medical complications between cohorts including DVT (OR 0.94, *p* = 0.6601) and PE (OR 0.80, *p* = 0.4102) (Table 3). Regarding surgery-related complications, the HRT cohort was associated with significantly higher 1-year incidences of dislocation (OR 1.35, *p* = 0.0269) and all-cause revision surgery (OR 1.23, *p* = 0.0105). The HRT cohort had a higher incidence of component exchanging revision surgery (OR 3.56, *p* < 0.0001), although no differences were noted between component retaining revision surgery rates (OR 1.10, *p* = 0.5635) (Table 4). There were no differences in rates of transfusion, UTI, pneumonia, AKI, periprosthetic fracture, prosthesis loosening, PJI, or wound disruption between HRT users and non-users (Tables 3 and 4).

**Table 2:** Patient demographics and comorbidities

		HRT (*n* = 3,936)	Control (*n* = 39,360)	OR	95% CI	P
Age	40–44	253	2426	1.05	0.91 – 1.20	0.5118
	45–49	443	4490	0.98	0.89 – 1.09	0.7741
	50–54	719	7031	1.03	0.94 – 1.12	0.5285
	55–59	858	8344	1.04	0.96 – 1.12	0.3807
	60–64	812	7911	1.03	0.95 – 1.12	0.4284
	65–69	572	5667	1.01	0.92 – 1.11	0.8186
	70–74	408	3916	1.05	0.94 – 1.17	0.4059
	75–79	151	1450	1.04	0.88 – 1.24	0.6290
	80 +	14	158	0.89	0.51 – 1.53	0.6638
Hypertension		1860	19,558	0.91	0.85—0.97	0.0036
Tobacco Use Disorder		653	7617	0.83	0.76—0.90	< 0.0001
Alcohol Use Disorder		84	843	1.00	0.79—1.25	0.9749
Diabetes Mellitus		583	6431	0.89	0.81—0.98	0.0132
Chronic Kidney Disease		122	1385	0.88	0.73—1.06	0.1716
Chronic Pulmonary Disease		753	8118	0.91	0.84—0.99	0.0269
Congestive Heart Failure		72	723	1.00	0.78—1.27	0.9729
Coronary Artery Disease		276	3156	0.87	0.76—0.98	0.026
Depression		1138	12,586	0.87	0.81—0.93	0.0001
Peripheral Vascular Disease		241	2488	0.97	0.84—1.11	0.6257

The number of patients within each age group were compared between cohorts using odds ratios and 95% confidence intervals. To compare rates of baseline medical comorbidities between cohorts, the number of patients diagnosed with each medical comorbidity prior to undergoing primary THA were also compared using odds ratios and 95% confidence intervals

**Table 3:** 90-day Medical Complications Following THA in HRT Users vs. Control

	HRT (*n* = 3936)		Control (*n* = 39,360)		OR	95% CI	P
DVT	52	1.32%	554	1.41%	0.94	0.70—1.25	0.6601
PE	15	0.38%	187	0.48%	0.80	0.47—1.36	0.4102
Transfusion	141	3.58%	1333	3.39%	1.06	0.89—1.26	0.5188
UTI	147	3.73%	1655	4.20%	0.88	0.74—1.04	0.1595
Pneumonia	32	0.81%	403	1.02%	0.79	0.55—1.14	0.207
AKI	35	0.89%	344	0.87%	1.02	0.72—1.44	0.922

Rates of 90-day medical complications were compared between cohorts using odds ratios and 95% confidence intervals

**Table 4:** 1-year Surgery-Related Complications Following THA in HRT Users vs. Control

	HRT (*n* = 3936)		Control (*n* = 39,360)		OR	95% CI	P
Dislocation	63	1.60%	469	1.19%	**1.35**	**1.03—1.76**	**0.0269**
Periprosthetic Fracture	40	1.02%	411	1.04%	0.97	0.70—1.35	0.8692
Loosening	35	0.89%	252	0.64%	1.39	0.98—1.99	0.0677
PJI	64	1.63%	735	1.87%	0.87	0.67—1.12	0.2838
All-cause Revision	173	4.40%	1413	3.59%	**1.23**	**1.05—1.45**	**0.0105**
Acetabular and/or femoral component revision	132	3.35%	1040	2.64%	**3.56**	**2.92 – 4.36**	** < 0.0001**
Acetabular and femoral component retaining revision	41	1.04%	373	0.95%	1.10	0.80 – 1.52	0.5635
Wound disruption	39	0.99%	417	1.06%	0.93	0.67—1.30	0.6878

Rates of 1-year surgery-related complications were compared between cohorts using odds ratios and 95% confidence intervals

Data regarding postoperative anticoagulation regimens were available for 2,104 patients in the HRT cohort and 20,485 patients in the control cohort (Table 5). HRT users were more likely to be prescribed warfarin (OR 1.21, *p* = 0.0001) or enoxaparin (OR 1.18, *p* = 0.0022) and less likely to be prescribed rivaroxaban (OR 0.62, *p* < 0.0001) compared to non-users. There were no differences in rates of aspirin or apixaban utilization postoperatively.

**Table 5:** Postoperative Anticoagulation Regimens following THA in HRT Users vs. Control

	HRT (*n* = 2104)		Control (*n* = 20,485)		OR	95% CI	P
Aspirin	483	22.96%	4431	21.63%	1.08	0.97—1.20	0.1605
Rivaroxaban	400	19.01%	5613	27.40%	**0.62**	**0.56—0.70**	** < 0.0001**
Apixaban	87	4.13%	795	3.88%	1.07	0.85—1.34	0.5667
Enoxaparin	489	23.24%	4180	20.41%	**1.18**	**1.06—1.31**	**0.0022**
Warfarin	645	30.66%	5466	26.68%	**1.21**	**1.10—1.34**	**0.0001**

Postoperative anticoagulation regimens prescribed within 7 days of primary THA obtained through pharmacy claims data available in the PearlDiver Mariner dataset

## Discussion

Our study revealed that female patients taking HRT were not found to be at increased risk for postoperative thromboembolism compared to female patients not taking HRT. Interestingly, women taking HRT were at an increased risk of sustaining a dislocation or undergoing revision surgery. The association between HRT and surgery-related complications following THA remains poorly understood and warrants further investigation.

We found no association between HRT use and the development of thromboembolism postoperatively. The pro-thrombotic effects of HRT are well described in the literature with multiple randomized clinical trials demonstrating an increased risk of idiopathic thrombosis formation among HRT users compared to non-users [6, 17, 25]. Our results are consistent with previously published studies investigating the risk of thrombosis among users of HRT or oral contraceptive therapy following various orthopaedic surgeries. Stone et al. conducted a national database study demonstrating no association between oral contraceptive use and venous thromboembolism following arthroscopic shoulder surgery [24]. Krych et al. performed a retrospective review of a single-center surgical database to determine risk factors for venous thromboembolism following arthroscopic knee surgery and found that HRT or oral contraceptive therapy increased the risk of postoperative venous thromboembolism although this result was only significant in a univariate analysis (OR 4.07, *p* = 0.04), and the difference became statistically insignificant when accounting for confounders with multivariate logistic regression (*p* = 0.09) [10]. While these results should only be cautiously extrapolated to arthroplasty procedures, a previous case–control study by Hurbanek et al. found no increased risk of venous thromboembolism following hip or knee arthroplasty among women taking HRT or selective estrogen receptor modulators [7]. Our findings support their conclusions that the thrombogenic effect of HRT is likely insignificant compared to the baseline risk of thrombosis following a major orthopaedic surgery. In comparing the HRT and control cohorts of the current study, rates of thromboembolism following THA were likely influenced by differences in anticoagulation regimens noted in pharmacy claims data (Table 5). Patients taking HRT at the time of THA were more likely to be prescribed warfarin (OR 1.21, *p* = 0.0001) or enoxaparin (OR 1.18, *p* = 0.0022) compared to non-users (Table 5). These results likely indicate that physicians are more aggressive while prescribing anticoagulants to HRT users due to the known increased baseline risk of thrombosis [4, 6, 8], although there are currently no guidelines in place regarding perioperative discontinuation of HRT therapy or altering postoperative anticoagulation regimens in this patient population. Surgeons would benefit from the development of a defined protocol to guide perioperative medical management among HRT users undergoing THA.

The association between the antiresorptive effects of HRT and revision surgery following arthroplasty procedures is poorly understood. There is a substantial body of evidence supporting the efficacy of bisphosphonate therapy for reducing periprosthetic bone loss, implant migration, aseptic loosening, and revision surgery [11, 16, 21], and the antiresorptive properties of HRT may similarly be protective against these complications. A previously published case–control study utilizing the UK General Practice Research Database found a strong protective effect of HRT against revision surgery but only if the HRT was initiated postoperatively (HR 0.24, *p* = 0.001) [15]. This protective effect was not found among HRT users who initiated therapy preoperatively (HR 1.06, *p* = 0.8) [19]. In the current study, the test cohort of patients were defined by preoperatively filled HRT prescriptions. While we found no statistically significant difference in rates of loosening, periprosthetic fracture, or PJI, the rates of component exchanging revision surgery (OR 3.56, *p* < 0.0001) and all-cause revision surgery (OR 1.23, *p* = 0.0105) were higher in the HRT cohort. These findings were likely influenced by an increased rate of dislocation among HRT users (OR 1.35, *p* = 0.0269). Our findings suggest that any estrogen-mediated alterations in bone metabolism that would facilitate osseointegration or decrease surgery-related complications may not be present if HRT is initiated preoperatively. Further research is warranted to better delineate how the timing and duration of HRT use may impact rates of surgery-related complications.

There are several limitations to the current study. As a retrospective database study, results are dependent upon accurate diagnosis of medical and surgery-related complications. Though a limitation of using a large administrative database is the possibility of inaccurate coding of diagnoses and procedures, the incidence of inaccuracy is estimated to be less than 1% [12]. Actual rates of DVT may be higher than reported in the current study as it is difficult to quantify the rate of undiagnosed DVT. Pharmacy insurance claims were utilized to determine which patients would be allocated to the HRT cohort, so we were unable to evaluate what percentage of patients may have discontinued HRT use in the perioperative period. Pharmacy insurance claims were also utilized to compare postoperative anticoagulation regimens. There were likely patients taking over the counter aspirin who were not accounted for in this analysis. While the HRT cohort was matched to a control cohort based on several comorbidities known to be risk factors for thrombosis including tobacco use, smoking is a relative contraindication to HRT. There were significantly fewer tobacco users in the HRT cohort (OR 0.83, *p* < 0.0001), and this may have acted as a confounder. Finally, these results were generated from a database of patients treated in the United States. Anticoagulant prescribing patterns following arthroplasty procedures vary geographically, and our findings should be cautiously extrapolated to other patient populations.

A strength of this study is the large sample size that allowed us to evaluate complications with low incidences. However, this produced some statistically significant results despite having small differences in complication rates between cohorts. Patients on HRT were more likely to sustain a dislocation (OR 1.35, 95% CI 1.03—1.76) or undergo revision surgery (OR 1.23, 95% CI 1.05—1.45), although this mildly increased risk is perhaps clinically inconsequential. The more significant findings of this study are likely the lack of increased thromboembolism risk among HRT patients and the differences in postoperative anticoagulation regimens noted between cohorts. These results identify a need for future research to determine how best to manage this population of patients. Another strength of this study is that patients included would have received treatments from a wide variety of surgeons and centers, making our results broadly applicable to clinical practice.

HRT use among patients undergoing THA is not associated with an increased rate of thromboembolism, although it did affect rates of surgery-related complications with higher incidences of dislocation and revision surgery among HRT users compared to non-users. Further research is warranted to better understand the association between HRT use and these surgery-related complications and to determine the ideal perioperative management of these medications.

## Data Availability

All data included in this study is commercially available.

## Abbreviations

THA: Total hip arthroplasty
DVT: Deep vein thrombosis
HRT: Hormone replacement therapy
ICD: International Classification of Diseases
CPT: Current Procedural Terminology
PE: Pulmonary embolism
UTI: Urinary tract infection
AKI: Acute kidney injury
PJI: Periprosthetic joint infection
OR: Odds ratio

## References

[1] Anderson DR, Morgano GP, Bennett C, Dentali F, Francis CW, Garcia DA, et al. (2019) American Society of Hematology 2019 guidelines for management of venous thromboembolism: prevention of venous thromboembolism in surgical hospitalized patients. Blood Adv. 3898–3944. 10.1182/bloodadvances.2019000975 10.1182/bloodadvances.2019000975 PMC6963238 31794602

[2] Bernhard ME, Barnes CL, DeFeo BM, Kaste SC, Wang X, Lu Z (2021). Total Hip Arthroplasty in Adolescents and Young Adults for Management of Advanced Corticosteroid-Induced Osteonecrosis Secondary to Treatment for Hematologic Malignancies. J Arthroplasty.

[3] Curtis EM, Moon RJ, Dennison EM, Harvey NC, Cooper C (2016). Recent advances in the pathogenesis and treatment of osteoporosis. Clin Med.

[4] Daly E, Vessey MP, Hawkins MM, Carson JL, Gough P, Marsh S. (1996) Risk of venous thromboembolism in users of hormone replacement therapy. Lancet. 977–980. 10.1016/s0140-6736(96)07113-9 10.1016/S0140-6736(96)07113-9 8855852

[5] Gialeraki A, Valsami S, Pittaras T, Panayiotakopoulos G, Politou M (2018). Oral Contraceptives and HRT Risk of Thrombosis. Clin Appl Thromb Hemost.

[6] Grady D, Wenger NK, Herrington D, Khan S, Furberg C, Hunninghake D, et al. (2000) Postmenopausal Hormone Therapy Increases Risk for Venous Thromboembolic Disease: The Heart and Estrogen/progestin Replacement Study. Obstet Gynecol Survey. 699–702. 10.1097/00006254-200011000-00021 10.7326/0003-4819-132-9-200005020-00002 10787361

[7] Hurbanek JG, Jaffer AK, Morra N, Karafa M, Brotman DJ (2004). Postmenopausal hormone replacement and venous thromboembolism following hip and knee arthroplasty. Thromb Haemost.

[8] Jick H, Derby LE, Myers MW, Vasilakis C, Newton KM. (1996) Risk of hospital admission for idiopathic venous thromboembolism among users of postmenopausal oestrogens. Lancet. 981–983. 10.1016/s0140-6736(96)07114-0 10.1016/S0140-6736(96)07114-0 8855853

[9] Kahlenberg CA, Garvey MD, Blevins JL, Sculco TP, Sculco PK, Figgie MP (2021). High Satisfaction and Activity Levels After Total Hip Arthroplasty in Patients Under Age 21. J Arthroplasty.

[10] Krych AJ, Sousa PL, Morgan JA, Levy BA, Stuart MJ, Dahm DL (2015). Incidence and Risk Factor Analysis of Symptomatic Venous Thromboembolism After Knee Arthroscopy. Arthroscopy.

[11] McDonald CL, Lemme NJ, Testa EJ, Aaron R, Hartnett DA, Cohen EM (2022). Bisphosphonates in Total Joint Arthroplasty: A Review of Their Use and Complications. Arthroplast Today.

[12] Medicare Medicaid Services. (2012) Medicare fee-for-service 2012 improper payments report. Payments Report. https://www.cms.gov/Research-Statistics-Data-and-Systems/Monitoring-Programs/Medicare-FFS-Compliance-Programs/CERT/Downloads/Medicare-Fee-for-Service-2012-Improper-Payments-Report.pdf. Accessed 10 Jan 2023

[13] Metcalfe D, Peterson N, Wilkinson JM, Perry DC. (2018) Temporal trends and survivorship of total hip arthroplasty in very young patients: a study using the National Joint Registry data set. Bone Joint J. 100-B: 1320–1329. 10.1302/0301-620X.100B10.BJJ-2017-1441.R2 30295530

[14] Mundi R, Axelrod DE, Najafabadi BT, Chamas B, Chaudhry H, Bhandari M (2020). Early Discharge After Total Hip and Knee Arthroplasty—An Observational Cohort Study Evaluating Safety in 330,000 Patients. J Arthroplasty.

[15] Prieto-Alhambra D, Javaid MK, Judge A, Maskell J, Cooper C, Arden NK (2015). Hormone replacement therapy and mid-term implant survival following knee or hip arthroplasty for osteoarthritis: a population-based cohort study. Ann Rheum Dis.

[16] Ro DH, Jin H, Park J-Y, Lee MC, Won S, Han H-S (2019). The use of bisphosphonates after joint arthroplasty is associated with lower implant revision rate. Knee Surg Sports Traumatol Arthrosc.

[17] Rossouw JE, Anderson GL, Prentice RL, LaCroix AZ, Kooperberg C, Stefanick ML (2002). Risks and benefits of estrogen plus progestin in healthy postmenopausal women: principal results From the Women’s Health Initiative randomized controlled trial. JAMA.

[18] Sandset PM (2013). Mechanisms of hormonal therapy related thrombosis. Thromb Res.

[19] Seeman E (2004). Estrogen, androgen, and the pathogenesis of bone fragility in women and men. Curr Osteoporos Rep.

[20] Shahi A, Chen AF, Tan TL, Maltenfort MG, Kucukdurmaz F, Parvizi J (2017). The Incidence and Economic Burden of In-Hospital Venous Thromboembolism in the United States. J Arthroplasty.

[21] Shi J, Liang G, Huang R, Liao L, Qin D (2018). Effects of bisphosphonates in preventing periprosthetic bone loss following total hip arthroplasty: a systematic review and meta-analysis. J Orthop Surg Res.

[22] Skeith L, Le Gal G, Rodger MA (2021). Oral contraceptives and hormone replacement therapy: How strong a risk factor for venous thromboembolism?. Thromb Res.

[23] Sloan M, Premkumar A, Sheth NP. (2018) Projected Volume of Primary Total Joint Arthroplasty in the U.S., 2014 to 2030. JBJS.100:1455. 10.2106/JBJS.17.01617 30180053

[24] Stone AV, Agarwalla A, Gowd AK, Jacobs CA, Macalena JA, Lesniak BP (2019). Oral Contraceptive Pills Are Not a Risk Factor for Deep Vein Thrombosis or Pulmonary Embolism After Arthroscopic Shoulder Surgery. Orthop J Sports Med.

[25] Vickers MR, MacLennan AH, Lawton B, Ford D, Martin J, Meredith SK, et al. (2007) Main morbidities recorded in the women’s international study of long duration oestrogen after menopause (WISDOM): a randomised controlled trial of hormone replacement therapy in postmenopausal women. BMJ. 335(7613):239. 10.1136/bmj.39266.425069.AD. Epub 2007 Jul 11. PMID: 17626056; PMCID: PMC1939792. 10.1136/bmj.39266.425069.AD PMC1939792 17626056

